# Helicobacter pylori in Bahrain: A Comprehensive Study of Patient Characteristics, Treatment Approaches, and Cure Rates

**DOI:** 10.7759/cureus.79341

**Published:** 2025-02-20

**Authors:** Mahmood Alawainati, Murtadha M Ali, Mohamed AlShaalan, Husain I Hasan, Qasim M Jasim, Safiya Almusawi

**Affiliations:** 1 Medicine, Royal College of Surgeons in Ireland, Manama, BHR; 2 Family Medicine, Primary Healthcare Centers, Manama, BHR; 3 Medical Microbiology, Salmaniya Medical Complex, Manama, BHR

**Keywords:** gastritis, helicobacter pylori, human, peptic ulcer, urea breath test

## Abstract

Introduction

*Helicobacter pylori* (*H. pylori*) bacteria is a major cause of gastritis, peptic ulcer disease, and functional dyspepsia. Although the treatment of *H. pylori* has evolved over time, challenges exist due to antibiotic resistance and variations in treatment outcomes. This study aimed to determine the characteristics and treatment outcomes of patients with *H. pylori* infection in Bahrain.

Materials and methods

A cross-sectional study of adults with *H. pylori* attending primary and secondary care settings was conducted. *H. pylori* was diagnosed based on the urea breath test. A computer-based simple random sample was obtained. Patients and treatment characteristics were determined from medical records. Descriptive and inferential analyses were conducted.

Results

A total of 709 patients were included, with an average age of 40.9±14.9 years. Nearly two-thirds of the participants were females (n=461, 65%) and diagnosed in primary care settings (n=464, 65.4%). Of the participants, 74.9% received treatment for *H. pylori*, with triple therapy (n=384, 72.3%) being the most used regimen in primary and secondary care hospitals, followed by the concomitant regimen (n=72, 13.6%). Bismuth-based quadruple therapy (P<0.001), concomitant therapy (P<0.001), and levofloxacin triple therapy (P=0.020) were used more frequently in secondary care settings compared to primary care settings. Nearly one-third of the patients underwent a test of cure (n=260, 36.7%). No significant association was found between the used regimen and the cure rate (P=0.938).

Conclusion

In summary, this study revealed comparable cure rates for *H. pylori* regardless of the antimicrobial regimens. Although most participants who underwent the test of cure had a negative test, the study highlighted the suboptimal management and follow-up of *H. pylori* cases. Prospective randomized controlled trials are needed to determine the exact effectiveness of each regimen among patients with *H. pylori*.

## Introduction

*Helicobacter pylori* (*H. pylori*) is a small, gram-negative, rod-shaped bacteria that infects nearly half of the world’s population [1]. It is a major cause of gastritis, peptic ulcer disease, and functional dyspepsia and has been implicated in the pathogenesis of gastric adenocarcinoma and mucosa-associated lymphoid tissue (MALToma) [2]. Therefore, accurate diagnosis and early management are important to avoid *H. pylori*-associated complications. Although *H. pylori* can affect all people, smokers, patients with low socioeconomic status, and patients with a positive family history of *H. pylori* are at a higher risk of having *H. pylori* [3,4].

Several diagnostic tests have been used to detect *H. pylori*, including histology-based tests, urea breath tests, stool antigen tests, and serology tests [5]. Although the choice of diagnostic test depends on the need for an upper endoscopy and local availability, urea breath tests, stool antigen tests, or endoscopy-based testing have higher accuracy for diagnosis and confirmation of eradication [6].

Multiple antibiotic regimens were tested to cure *H. pylori *infection. Studies were inconsistent regarding the efficacy of these regimens against *H. pylori* [7]. Some studies showed that all regimens exhibited comparable *H. pylori* eradication rates. For instance, several randomized controlled studies found similar success rates in eradicating *H. pylori* regardless of the regimen used [8,9]. In contrast, many studies reported variable efficacy levels of *H. pylori* treatments. A large systematic review of 68 interventional studies revealed that reverse hybrid therapy was superior to other regimens, while the triple therapy regimen was the least effective treatment in eradicating *H. pylori* [10]. Some guidelines showed that clarithromycin-based triple therapy has been replaced by bismuth quadruple therapy and concomitant therapy due to the increasing rates of clarithromycin resistance [11,12].

A major reason for this variation in the cure rates is the presence of antimicrobial resistance. Resistance rates vary by location depending on local antibiotic usage rates and differences in genetic and previous antibiotic usage. Some studies reported more than 50% resistance rates in clarithromycin-based regimens [13,14]. Therefore, several guidelines recommend assessing antibiotic cure rates and sensitivity profiles against *H. pylori*. Nonetheless, the exact resistance to clarithromycin and metronidazole-based regimens remains undetermined in the Arabian Gulf region [15].

Patient characteristics, including smoking and age, were also found to be predictors of *H. pylori* resistance in the literature [16]. In contrast, some studies reported no impact of sociodemographic characteristics on *H. pylori* cure rates [17]. In Bahrain, few studies were conducted to determine the antimicrobial susceptibility of *H. pylori*. An old study conducted in 2001 in Bahrain found that 57% and 33% of the strains were resistant to metronidazole and clarithromycin, respectively [18]. However, none of the studies addressed the characteristics of patients with *H. pylori* infections and their treatment outcomes. Therefore, this study aimed to determine the characteristics and treatment outcomes of patients diagnosed with *H. pylori* between August 2022 and August 2023 in all primary and secondary healthcare centers in Bahrain.

## Materials and methods

This cross-sectional analytical study was conducted in all governmental primary care centers (27 centers) and secondary hospitals in Bahrain between August 2022 and August 2023. Ethical approval was obtained from the Institutional Review Board (IRB) (no. 89150823 dated 15/8/2023).

Patients who had a positive urea breath test between June 2021 and June 2023 were eligible to be included. For patients with two positive tests, only the characteristics (i.e., the treatments and test of cure outcomes) of the first positive infection were included. A total of 2100 patients had a positive urea breath test in the selected period. To achieve a confidence level of 95% and a 5% margin of error, a sample size of 325 participants was needed. To increase the power of the study, a larger sample size was targeted. The sample was taken using a random computer-based technique.

A data collection tool was designed to obtain the data. The tool consisted of three parts: the demographic and baseline characteristics part, which included age, sex, nationality, and past medical history; the treatment characteristics part, which comprised the type of antibiotics, name of the proton pump inhibitor (PPI), dose, frequency, documented allergies, and post-treatment test results; and the data extraction part, which included the patient demographics, management, and clinical outcomes. Three trained physicians extracted data on patient demographics, management, and clinical outcomes from the medical records.

Untreated *H. pylori* was considered if no antibiotic regimen for *H. pylori* was documented in the electronic medical records, while treated *H. pylori* was considered if the antibiotic regimen was documented. Outcomes of *H. pylori* were assessed after six weeks of antibiotic use and were determined based on the urea breath test results.

Triple therapy was defined as a combination use of a PPI, amoxicillin, and clarithromycin/metronidazole; concomitant therapy regimen was defined as the use of a PPI, clarithromycin, amoxicillin, and metronidazole for 10-14 days, while bismuth quadruple therapy was defined as the use of a PPI, bismuth, tetracycline, and metronidazole for 10-14 days. In addition, levofloxacin triple therapy consisted of a PPI, levofloxacin, and amoxicillin for 10-14 days, and hybrid therapy consisted of a PPI and amoxicillin for seven days, followed by a PPI, amoxicillin, clarithromycin, and nitroimidazole for seven days.

Means with confidence intervals were calculated for continuous variables, while numbers and percentages were calculated for categorical variables. The Kolmogorov-Smirnov and the Shapiro-Wilk tests were conducted to assess the normality of data. As appropriate, the chi-square test, Fisher's exact test, and T-test were performed by an independent statistician to determine the differences between the different groups. A P-value of less than 0.05 indicated significant associations. IBM SPSS Statistics for Windows, Version 26 (Released 2019; IBM Corp., Armonk, New York) was used to analyze the data.

## Results

A total of 709 patients with positive urea breath tests were analyzed with an average age of 40.9±14.9 years. Nearly two-thirds of the cohort were females (n=461, 65%) and managed in primary care centers (n=464, 65.4%). Of the comorbidities, 20% (n=142) of the patients had essential hypertension, 17.2% (n=122) had dyslipidemia, and 13.4% (n=95) had diabetes mellitus. Table 1 presents the sociodemographic and baseline characteristics of the participants with *H. pylori*.

**Table 1:** Sociodemographic and baseline characteristics of the participants with H. pylori

Variable		n (%)
Nationality	Bahraini	640 (90.3)
	Non-Bahraini	69 (9.7)
Sex	Male	248 (35)
	Female	461 (65)
Age, mean± SD		40.9±14.9
Essential hypertension	Yes	142 (20)
	No	567 (80)
Dyslipidemia	Yes	122 (17.2)
	No	587 (82.8)
Diabetes mellitus	Yes	95 (13.4)
	No	614 (86.6)
Cardiac diseases	Yes	20 (2.8)
	No	689 (97.2)
Stroke	Yes	1 (0.1)
	No	708 (99.9)
Sickle cell disease	Yes	5 (0.7)
	No	704 (99.3)
Allergy status	Yes	12 (1.7)
	No	697 (98.3)
Origin	Primary care	464 (65.4)
	Secondary care	245 (34.6)

Of all cases with *H. pylori*, 74.9% received treatment, with triple therapy (n=384, 72.3%) being the most adopted, followed by concomitant (n=72, 13.6%) and bismuth-based regimens (n=36, 6.8%). More than 5% of the patients received a single antibiotic. Additionally, the test of cure was performed only in 260 patients (36.7%), and the cure rate of the patients who performed the test was as high as 62.3% (Table 2).

**Table 2:** Treatment regimen of patients with H. pylori in Bahrain *Single regimens: amoxicillin, amoxicillin-clavulanic acid, metronidazole, or clarithromycin alone

Variables		n (%)
Treatment status (TN=709)	Treated	531(74.9)
	Untreated	178(25.1)
Treatment regimen (TN=531)	Triple therapy	384(72.3)
	Concomitant therapy	72(13.6)
	Bismuth quadruple therapy	36(6.8)
	Levofloxacin triple therapy	9(1.7)
	Hybrid therapy	1(0.2)
	Other treatments*	29(5.5)
Test of cure (TN=709)	Done	260(36.7)
	Not done	449(63.3)
*Helicobacter pylori* outcome (TN=260)	Resolved (cured)	162(62.3)
	Persistent (uncured)	98(37.7)

**Figure 1:**
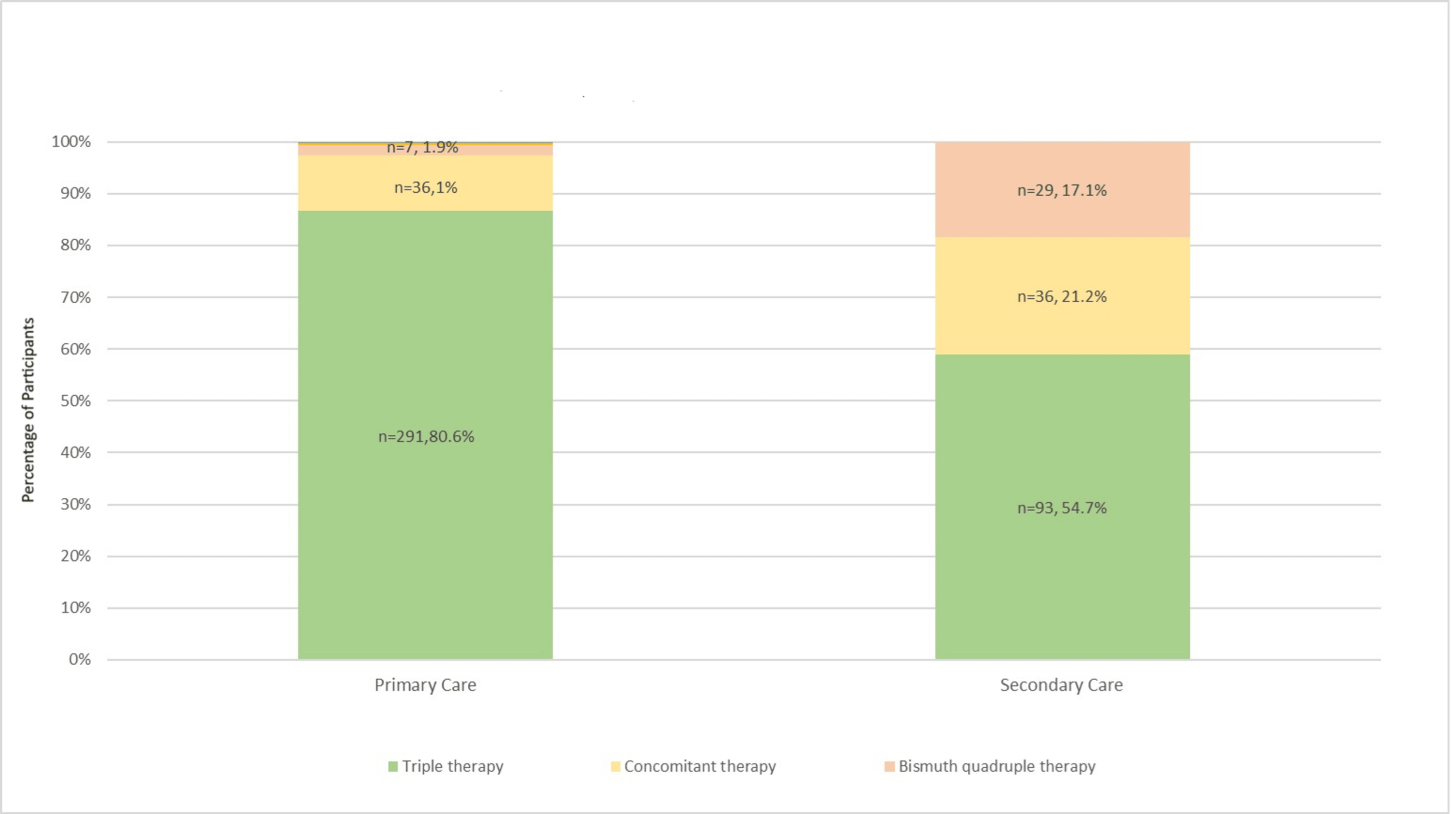
Treatment regimens of H. pylori in Bahrain

As shown in Figure 1, triple therapy was the most commonly used regimen in primary and secondary healthcare settings (n=80.6% and 21.2%).

Table 3 shows no differences in the sociodemographic characteristics and comorbidities of patients with *H. pylori* in primary and secondary care settings. Although the predominant regimen utilized was triple therapy in both primary and secondary care settings, bismuth-based quadruple therapy (P<0.001), concomitant therapy (P<0.001), and levofloxacin triple therapy (P=0.020) were used more frequently in secondary care settings compared to primary care settings.

**Table 3:** Association between treatment settings of Helicobacter pylori treatment, sociodemographic characteristics, comorbidities, and treatment regimens

Variables		Primary Care TN= 151	Secondary Care TN=109	Test Value	P-value
Nationality	Bahraini	141(57.8)	103(42.2)	0.137	0.711
	Non-Bahraini	10(62.5)	6(37.5)		
Sex	Male	49(60.5)	32(39.5)	0.282	0.595
	Female	102(57)	77(43)		
Age		41.1±15.6	43.42±13.7	4.071	0.207
Diabetes mellitus	Yes	21(51.2)	20(48.8)	0.940	0.332
	No	130(59.4)	89(40.6)		
Essential hypertension	Yes	34(60.7)	22(39.3)	0.204	0.652
	No	117(57.4)	87(42.6)		
Dyslipidemia	Yes	26(56.5)	20(43.5)	0.056	0.814
	No	125(58.4)	89(41.6)		
Cardiac diseases	Yes	6(75)	2(25)	0.971	0.474
	No	145(57.5	107(42.5)		
Sickle cell	Yes	2(50)	2(50)	0.109	0.559
	No	149(58.2)	107(41.8		
Triple vs. others	Triple	107(66.9)	53(33.1)	23.558	<0.001
	Single	27(62.8)	16(37.2)		
	Quadruple/Others	17(29.8)	40(70.2)		
Triple therapy	Yes	107(66.9)	53(33.1)	13.225	<0.001
	No	44(44)	56(56)		
Bismuth quadruple therapy	Yes	1(7.1)	13(92.9)	15.766	<0.001
	No	150(61)	96(39)		
Concomitant therapy	Yes	13(39.4)	20(60.6)	5.419	0.020
	No	138(60.8)	89(39.2)		
Levofloxacin triple therapy	Yes	2(22.2)	7(77.8)	3.367	0.037
	No	149(59.4)	102(40.6)		
Cured	Yes	93(57.4)	69(42.6)	0.079	0.778
	No	58(59.2)	40(40.8)		

No significant associations were found between treatment outcomes and baseline characteristics, comorbidities, and prescribed regimens of the participants (Table 4).

**Table 4:** Association between Helicobacter pylori cure status, sociodemographic characteristics, comorbidities, and treatment regimens

Variables		Cured, TN=162 n (%)	Not Cured, TN=98 n (%)	Test Value	P-value
Sex	Male	48(59.3)	33(40.7)	0.466	0.495
	Female	114(63.7)	65(36.3)		
Age		43.7±12.7	39.2±16.2	6.968	0.086
Diabetes mellitus	Yes	23(56.1)	18(43.9)	0.799	0.371
	No	139(63.5)	80(36.5)		
Hypertension	Yes	39(69.6)	17(30.4)	1.635	0.201
	No	123(60.3)	81(39.7)		
Dyslipidemia	Yes	29(63)	17(37)	0.013	0.910
	No	133(62.1)	81(37.9)		
Cardiac diseases	Yes	5(62.5)	3(37.5)	<0.001	0.649
	No	157(62.3)	95(37.7)		
Sickle cell diseases	Yes	3(75)	1(25)	0.279	0.598
	No	159(62.1)	97(37.9)		
Origin of the sample	Primary care	93(61.6)	58(38.4)	0.079	0.778
	Secondary care	69(63.3)	40(36.7)		
Triple vs. others	Single	26(60.5)	17(39.5)	0.053	0.938
	Triple	101(63.1)	59(36.9)		
	Quadruple/others	35(61.4)	22(38.6)		

## Discussion

This study aimed to determine the clinical features and management of *H. pylori *patients in Bahrain. The results revealed that most patients with *H. pylori* were females, seen in primary care settings, and received triple antimicrobial regimens. In addition, the results revealed that less than two-thirds of cases were cured after the first antibiotic regimen, and no relationship was found between the *H. pylori* cure rate and the regimen used.

The average age of participants in the present study is comparable to that of the previous studies, with a similar male-to-female ratio [19]. The literature revealed an inconsistent relationship between an individual’s sex and the risk of *H. pylori* infection [20]. While some studies suggested a higher prevalence of H*. pylori* among one sex, others reported no significant differences [21]. In this study, the majority of *H. pylori* infections were found in females, which supports the observation of a higher prevalence among women [22,23]. In contrast to the findings in this study, some studies, including a previous study conducted in Bahrain, reported higher rates of *H. pylori* among males compared to females [24,25]. The exact reasons for these differences remain unknown.

Most participants who underwent the test of cure (62.3%) exhibited a negative test result. Studies revealed variable eradication rates among patients with *H. pylori* ranging between 20% and 90% [26-28]. Consistent with the literature, the present study revealed no association between *H. pylori* eradication rates and patient characteristics, including age, sex, and comorbidities such as diabetes mellitus, hypertension, and dyslipidemia [29,30].

Furthermore, the influence of the used regimen against *H. pylori* on the cure rate was assessed. Although the present study revealed no association between the used regimen and the cure rate, such an association has been reported in the literature [26,27,29]. A possible reason for these inconsistencies includes variable antibiotic susceptibility across studies, as reported in some studies [27]. Additionally, most patients in the present study had not performed a test of cure, which could have influenced the overall cure rates. Nonetheless, some studies reported findings similar to those of the present paper [8,28].

This study has several strengths, including adopting a random sampling technique, recruiting patients from primary and secondary care settings, and assessing several patient and treatment characteristics. However, the study has some limitations as well. Not all patients received treatment and underwent a test of cure. In addition, the present study did not assess some factors, such as smoking, name of the PPI used, and medication adherence, despite their known impact on cure rates. Some patients could have received treatment from other private institutions, which could have underestimated the number of treated cases.

## Conclusions

In summary, this study revealed comparable cure rates against *H. pylori* regardless of the antimicrobial regimen used and a relatively good cure rate. Triple therapy remains the most used regimen to treat *H. pylori* in primary and secondary care settings in Bahrain. In addition, no association between the sociodemographic characteristics of the patients and the overall cure rates was found. Prospective randomized controlled trials are needed to determine the exact effectiveness of each regimen among patients with *H. pylori* in Bahrain. Further research is essential to explore the potential impact of antimicrobial resistance on treatment outcomes and guide future therapeutic strategies.
